# All-Electrochem-Active Graphite Electrode Enabled by Manipulating Li^+^ Activity of Inactive Components for High-Energy Batteries

**DOI:** 10.1021/acsaem.5c00794

**Published:** 2025-06-09

**Authors:** Junjin Zhang, Qitao Shi, Chen Lu, Xiangqi Liu, Jiaqi Wang, Cheng Zhang, Zhipeng Wang, Luwen Li, Alicja Bachmatiuk, Yanbin Shen, Ruizhi Yang, Mark H. Rümmeli

**Affiliations:** † Soochow Institute for Energy and Materials Innovation, College of Energy, Key Laboratory of Advanced Carbon Materials and Wearable Energy Technologies of Jiangsu Province, Key Laboratory of Core Technology of High Specific Energy Battery and Key Materials for Petroleum and Chemical Industry, 12582 Soochow University, Suzhou 215006, China; ‡ i-Lab, CAS Center for Excellence in Nanoscience, Suzhou Institute of Nano-Tech and Nano-Bionics (SINANO), Chinese Academy of Sciences (CAS), Suzhou 215123, China; § School of Electronic and Information Engineering, Changshu Institute of Technology, Changshu 215500, China; ∥ Faculty of Chemistry, Wroclaw University of Science and Technology, Wybrzeze Wyspiarskiego 27, Wroclaw 50-370, Poland; ⊥ Soochow Institute for Energy and Materials Innovation, College of Energy, Key Laboratory of Advanced Carbon Materials and Wearable Energy Technologies of Jiangsu Province, Soochow University, Suzhou 215006, China; # Jiangsu Key Laboratory of Advanced Negative Carbon Technologies Soochow University, Suzhou 215123, China

**Keywords:** all-electrochem-active, Li^+^ activity manipulating, graphite electrode, MXene, high-energy batteries

## Abstract

Graphite anodes have approached their theoretical specific capacity of 372 mA h g^–1^, which becomes an obstacle for further increasing the energy density of commercial lithium-ion batteries. Various strategies have been proposed to enhance the energy density of graphite-based full batteries, such as decreasing the usage of inactive binders and conductive additives and exploring graphite/SiO_*x*_ composite anodes. Nevertheless, the anodes cannot balance energy density, power density, and cycling stability. In this study, we designed an all-electrochem-active graphite electrode by manipulating the Li^+^ activity of the inactive components to improve the energy density of the entire electrode. In our study, colloidal two-dimensional titanium carbide nanosheets (MXene) were employed as binders, and carbon-coated titanium dioxide nanoparticles with oxygen defects (TiO_2–*x*_@C) acted as conductive additives in the electrode configurations. Both MXene and TiO_2–*x*_@C can function as active materials to store lithium ions by reversible insertion and extraction with little electrochemical degradation. As a result, the all-electrochem-active graphite electrodes demonstrated a superior specific capacity of 394 mA h g^–1^ at a current density of 0.2C after 300 cycles. This concept of all-electrochem-active electrodes is anticipated to inspire future research on high-energy-density batteries by activating the Li^+^ affinities of binders and conductive additives.

## Graphical abstract

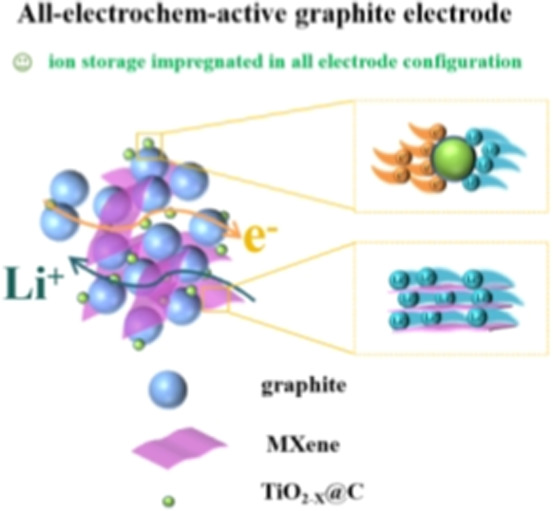

## Introduction

With the continuous development of portable mobile devices and new-energy vehicles, the development of high-energy-density lithium-ion batteries has been promoted.^1–3^ At the same time, higher requirements have been put forward for battery performance, which require the development of high-energy-density batteries. At present, the theoretical specific capacity of commercial graphite anodes has reached 372 mA h g^–1^, which has become an obstacle to further improving the energy density of commercial lithium-ion batteries. Various strategies have been proposed to improve the overall energy density of full batteries with carbon-based anodes, including reducing the use of inactive binders and conductive additives. For example, Ahn et al. introduced a multifunctional poly­(melamine-*co*-formaldehyde) (MF copolymer) additive, which produced a relatively more fluid and well-dispersed slurry using only 0.08 wt %, to prepare a cylindrical battery with a highly flexible high-energy-density electrode (active material load >98.4%).^4^ Additionally, replacing graphite with porous carbon or graphite/SiO_*x*_ composite materials with a thin Si layer coating is a common method to improve the energy density of carbon-based anode batteries.^5–7^ However, these anodes do not strike a good balance between energy density, power density, cycle stability, and production costs. Therefore, an equally significant aspect is the development of electrode additives, which are necessary to maintain the conductive network and mechanical integrity of the electrodes.

Electrode additives include binders and conductive additives. Generally, binders mechanically bond the active material and conductive additives to the current collectors during the cycling process, and the conductive additives ensure electron transfer throughout the electrode.^8–10^ Nevertheless, the binders and conductive additives are not electrochemically active. Nonelectrochemically active ingredients make almost no contribution to the capacity of the electrode, thereby lowering its energy density. Therefore, the removal of nonelectrochemically active components can increase the electrochemical capacity and mass energy density of the overall electrode.

Thus, endowing electrode additives with high Li^+^ activity is a potential approach for enhancing the energy density of graphite electrodes. Therefore, electrode additives are required to maintain structural and electrochemical stability upon repeated lithiation and delithiation at a relatively low-voltage plateau. Consequently, insertion of anode materials is a promising approach. Specifically, insertion materials with low dimensions and high surface areas can be used as binders to build sufficient mechanical connections between the active materials, conductive additives, and current collectors after facile optimization.^11,12^ In addition, nano structuring, defect engineering, and carbon coating of insertion materials are widely reported strategies for improving bulk electronic conductivity.^13–19^

Herein, we constructed an all-electrochemically active graphite electrode by employing two-dimensional colloidal MXene nanosheets as binders and carbon-coated TiO_2_ with oxygen defects as conductive additives, without the presence of nonelectrochemically active polymer binders and conductive additives. The bulk flexibility and surface hydroxyl groups of colloidal MXene nanosheets favor mechanical connections to facilitate the formation of electrodes, while defect engineering and graphitic coating of nanosized TiO_2_ particles (TiO_2–*x*_@C) endow excellent electronic conductivity that is even higher than that of commercial carbon black; thus, TiO_2–*x*_@C can produce a continuous conductive network that efficiently distributes and transports charges. Simultaneously, MXene and TiO_2–*x*_@C can reversibly store lithium ions without severe structural and electrochemical degradation. Therefore, the electrochemically active graphite electrode provided storage sites for lithium ions over the entire electrode area, which improved the energy density of the graphite electrode. The removal of inactive additives also promotes lithium-ion transport in the electrode microstructure, improving the capacity retention at the lifting cycling rate.

## Results and Discussion

### Design Strategies of All-Electrochem-Active Graphite Electrode

Figure 1 illustrates the design strategy for the all-electrochem-active graphite electrode. The left panel shows a typical graphite electrode comprising graphite, polymer binders, and conductive nanocarbon additives. Polymer binders and conductive additives are incapable of conducting electrons or lithium ions, thereby lowering the overall energy density of the electrode. The right panel in Figure 1 depicts the configuration of an all-electrochemically active graphite electrode with hydroxy-enriched colloidal MXene nanosheets as binders, nanosized TiO_2_ with oxygen defects, and carbon coating (TiO_2–*x*_@C) as conductive additives. In the electrode configuration, MXene binds graphite and TiO_2–*x*_@C to the current collector well, which provides good mechanical reinforcement for the electrode; the presence of TiO_2–*x*_@C enables the electrode to form a continuous conductive network and realize fast charge transfer. MXene and TiO_2–*x*_@C both have a strong activity for lithium ions and electrons, realizing their potential to reversibly store lithium ions in the electrode, thus improving the energy and power densities.

**Figure 1:**
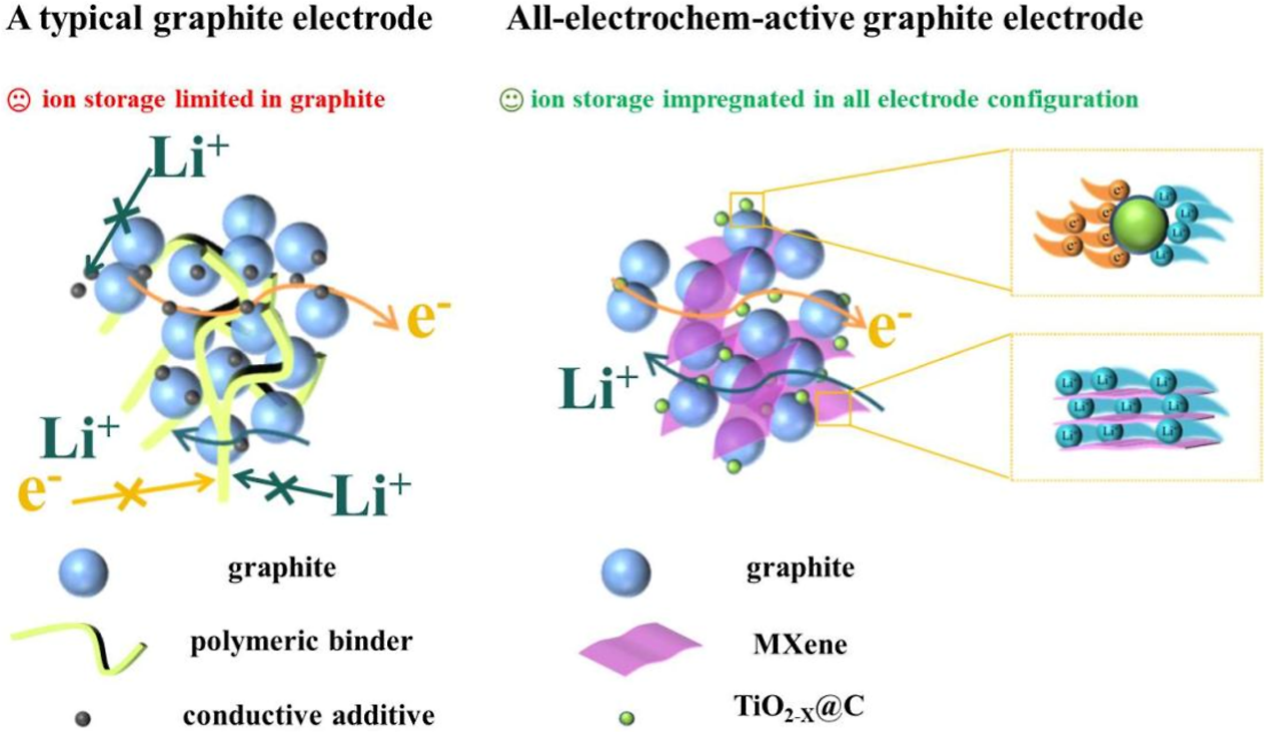
Schematic depicting a typical electrode (left) and the all-electrochem-active graphite electrode (right), with inset schemes showing transport of lithium ions in the TiO_2–*x*_@C conductive additives (top) and MXene binders (bottom). For detailed morphological characterization, please refer to the SEM images in Figure S1.

### Physicochemical Characterization of MXene Binders

Colloidal Ti_3_C_2_T_*X*_ MXenes were prepared by traditional LiF/HCl etching, repeated centrifugation, and direct collection in the colloidal state without freeze-drying. Figure 2a shows the viscosities of the as-prepared colloidal MXenes. The SEM results showed that multilayer Ti_3_AlC_2_ (Figure S2) was successfully etched into single-layer MXene (Figure 2b). In contrast, the MXene sheets tended to agglomerate after freeze-drying (Figure S3), resulting in a decreased adhesion strength. The single-layer structure of MXene was further confirmed using TEM (Figure 2c). The XRD pattern (Figure 2d) shows that the Al peak of Ti_3_AlC_2_ disappears after HCI and LiF etching, and the diffraction peak of (002) moves to a small angle (2θ moves from 9.64° to 6.76°), demonstrating the successful preparation of MXene. The Raman spectra of MXene present three distinct peaks at 206 cm^–1^ ascribed to the vibrational anatase phase, and at 400 cm^–1^ and 593 cm^–1^ corresponding to the rutile phase (Figure S4).^20^

**Figure 2:**
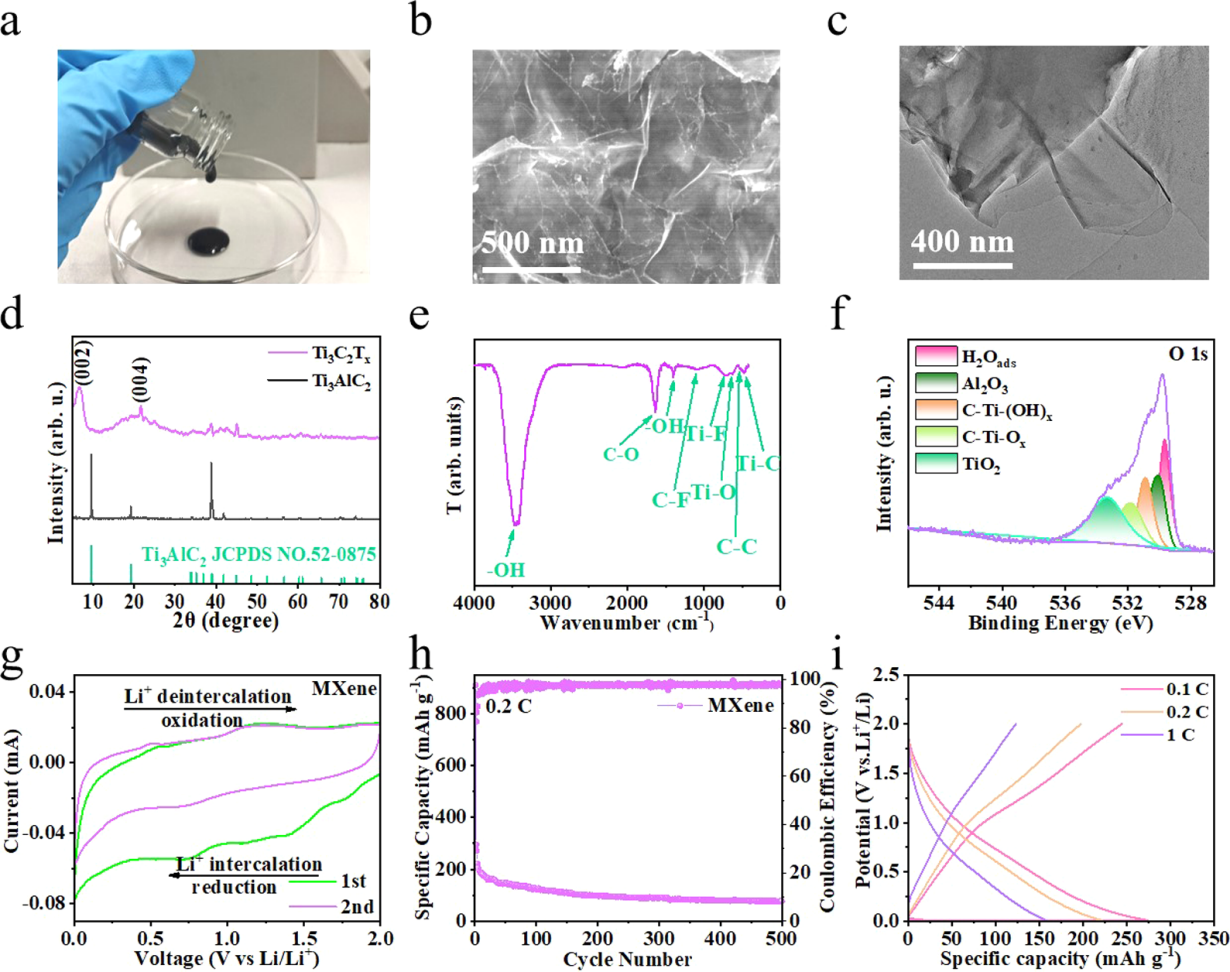
(a) Optical image, (b) SEM image, (c) TEM image, (d) XRD pattern, (e) FTIR spectrum, and (f) O 1s XPS spectrum of MXene. (g) CV of the MXene electrode at a scan rate of 0.2 mV s^–1^. (h) Cycling performance of the MXene electrode at 0.2C. (i) Voltage-capacity curves of MXene electrode at different current densities.

Fourier transform infrared (FTIR) spectroscopy was carried out to analyze the functional groups on the MXene surface. The FTIR spectrum of MXene (Figure 2e) shows a sharp –OH peak of high intensity at 3600–3200 cm^–1^ along with a weak –OH peak at 1500–1300 cm^–1^, which elucidates the origin of hydrogen bonding-based adhesion capability. Additionally, various carbon and titanium bond vibrations were clearly observed in the FTIR spectrum.^21^ The freeze-dried Ti_3_C_2_T_*X*_ MXene shows a high specific surface area of 48.3 m^2^ g^–1^ (Figure S5). According to the SEM analysis of colloidal and freeze-dried MXene, it can be speculated that colloidal MXene has a much larger specific surface area than freeze-dried MXene, which favors long-range connection and adhesion inside the electrode configuration.

The surface chemical properties of the MXenes were characterized using X-ray photoelectron spectroscopy (XPS), and their terminal functional groups were determined. The elemental composition of MXene is shown in Figure S6. The C 1s spectrum of the MXene sample shows three peaks at 282.0, 284.9, and 289.2 eV (Figure S7a), corresponding to C–Ti–T_*x*_, C–C, and C–O, respectively. And the F 1s spectrum of MXene shows three peaks at 685.1, 686.1, and 686.6 eV (Figure S7b), corresponding to C–Ti–F_*X*_, Al–F, and LiF, respectively. In Figure 2f, the five peaks in the O 1s spectrum at 529.7, 530.1, 530.9, 531.9, and 533.3 eV were fitted, corresponding to H_2_O_ads_, Al_2_O_3_, C–Ti–(OH)_*x*_, C–Ti–O_*x*_, and TiO_2_ respectively.^22^ The XPS results showed the presence of –O, –F, and –OH terminal groups on the MXene surface. The polar –OH group can form hydrogen bonds with water molecules, which increases the intermolecular interaction force and improves the viscosity of the viscous MXene ink.^23^

Cyclic voltammetry was performed to determine the electrochemical activities of the as prepared MXenes. The cyclic voltammogram (CV) of the MXene electrode displays two recognized sets of oxidation and reduction peaks corresponding to the two-layer spacing, where Li^+^ intercalation (deintercalation) occurred sequentially between the MXene layers (Figure 2g).^24^ The stable 97% Coulombic efficiency observed for the MXene electrode at 0.2C reveals the reversible lithium-ion insertion/extraction behavior at the electrode/electrolyte interface (Figure 2h). Additionally, the charge and discharge curves at different current densities show satisfactory rate capability of MXene as an active material (Figure 2i). In conclusion, MXene has the ability to store lithium ions.

### Physicochemical Characterization of TiO_2–*x*_@C Conductive Additives

Figure 3a illustrates the morphology of the TiO_2–*x*_@C particles, which had an average diameter of approximately 66 nm (Figure S9b). The TEM image shows that the TiO_2–*x*_@C observed by SEM consisted of multiple smaller TiO_2–*x*_@C units with sizes close to those of the as-purchased TiO_2_ (Figure 3b). This nanosized morphology is conducive to the construction of more point-to-point conductive connections. The high-resolution TEM (HRTEM) image shows that carbon was fully coated on TiO_2–*x*_ through the CVD process (Figure S10), which is the key to enhancing its electrical conductivity. In addition, the lattice spacing of the (112), (208), and (213) crystal facets and the (215) crystal facet of the TiO_2_ (Anatase) phase are demonstrated in the HRTEM image, as well as in the SAED diagram (Figure S11). The scanning transmission electron microscopy (STEM) image and corresponding EDX mapping show a uniform distribution of Ti, O, and C, further confirming that C was uniformly coated on TiO_2–*x*_ (Figure 3c).

**Figure 3:**
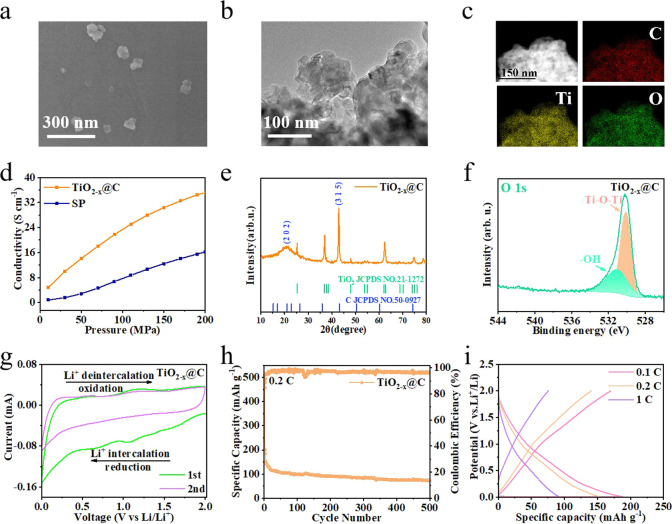
(a) SEM image, (b) TEM image, (c) STEM image, and corresponding elemental mappings of TiO_2–*x*_@C. (d) Electrical conductivity tests of TiO_2–*x*_@C and commercial carbon black SP. (e) XRD pattern and (f) O 1s XPS spectrum of TiO_2–*x*_@C. (g) CV of the TiO_2–*x*_@C electrode at a scan rate of 0.2 mV s^–1^. (h) Cycling performance of the TiO_2–*x*_@C electrode at 0.2C. (i) Voltage-capacity curves of the TiO_2–*x*_@C electrode at different current densities.

The electrical conductivity of TiO_2–*x*_@C was significantly enhanced by the carbon coating, as shown in Figure 3d. When the pressure applied to TiO_2–*x*_@ reaches 200 MPa, its electrical conductivity reaches 35 S cm^–1^, which is higher than that of commercial carbon black SP by 17 S cm^–1^. Excellent electrical conductivity ensures electron transport in the all-electrochemically active graphite electrode. To further analyze the structure of TiO_2–*x*_@C, XRD was performed (Figure 3e). In addition to the TiO_2_ (anatase) structure, two peaks at (202) and (315) were ascribed to the carbon coating. In the Raman spectrum of TiO_2–*x*_@C (Figure S12), significant signals at 143, 418, 515, and 610 cm^–1^ are characteristic of TiO_2_. Moreover, a typical D band (1345 cm^–1^) attributed to disordered carbon and a G band (1590 cm^–1^) attributed to the sp^2^ carbon structure were detected. The value of *I*_D_/*I*_G_ was 0.94, implying many defects within the coated carbon, which can promote ion diffusion and enhance reaction kinetics.^25^

XPS measurements were conducted to further understand the chemical composition of TiO_2–*x*_@C. The survey XPS spectrum of TiO_2–*x*_@C is shown in Figure S13b. The O 1s high-resolution spectrum (Figure 3f) shows two peaks at the binding energies of 529.9 eV (Ti–O–Ti) and 531.5 eV (−OH). The relative content of –OH in TiO_2–*x*_@C was higher than that in TiO_2_, indicating the presence of oxygen defects in TiO_2–*x*_@C. The Ti 2p spectrum of TiO_2–*x*_@C (Figure S14) consists of four peaks. The peaks located at 464.1 and 458.8 eV represent Ti^4+^, and the peaks centered at 463.9 and 455.4 eV represent Ti^3+^. In contrast, no Ti^3+^ peaks are observed in the Ti 2p spectrum of TiO_2_ (Figure S15a), proving that reduction sintering converts Ti^4+^ to Ti^3+^ owing to oxygen defects in the TiO_2_ nanocrystals.^26^

Electron paramagnetic resonance (EPR) spectroscopy was performed to confirm the presence of O-vacancy (Figure S16). All samples showed a signal at *g* = 2.003, with large differences in peak intensity owing to the different amounts of Ti^3+^ and oxygen vacancies in TiO_2_ and TiO_2–*x*_@C.^27^ TiO_2–*x*_@C exhibited a high peak intensity, which further indicated that there was a high concentration of O vacancies in TiO_2–*x*_@C. The generation of defects (vacancies) can effectively reduce the bandgap of TiO_2_, thus improving the conductivity and reducing the ion diffusion barrier.^28,29^

In the CV of the TiO_2–*x*_@C electrode, the reduction peak at 1.7 V and oxidation peak at 2.0 V respectively represent the insertion and extraction of Li^+^ in TiO_2_ (Figure 3g).^30^ The TiO_2–*x*_@C electrode demonstrated a stable Coulombic efficiency of 96% at 0.2C, indicating reversible Li^+^ insertion/extraction at the electrode/electrolyte interface (Figure 3h). Additionally, the charge and discharge curves at different current rates show the reversibility of lithium storage by TiO_2–*x*_@C at different current densities (Figure 3i). TiO_2–*x*_@C has the ability to store lithium ions and builds fast electron-conducting paths inside the electrode.

### Electrochemical Performance

To better confirm the advantages of the as-designed all-electrochem-active graphite electrode (denoted as AEA-G), we systematically compared its electrochemical behavior with conventional graphite electrodes prepared with commercial PVDF binders/SP conductive additives (denoted as PS-G). The CVs of the AEA-G anodes were obtained to comprehensively understand their electrochemical behavior (Figure 4a). During the reduction reaction of cathodic scanning, cathodic peaks appear at about 0.01 and 0.14 V. During anode scanning, the anode peak appeared at approximately 0.24 V. These peaks correspond to the embedding and extraction of Li ions in the graphite layer. This can also be attributed to classical LiCx phase transitions.^31^ In general, the oxidation peaks of the AEA-G electrode are sharp, indicating fast reaction kinetics. The redox peak potential difference of the AEA-G electrode was smaller than that of the PS-G electrode (Figure S17), indicating that the electrode polarization of the AEA-G electrode was lower than that of the PS-G electrode, which is favorable for the rate performance.^32^ The higher oxidation peak intensity of the AEA-G electrode confirms this.^33^

**Figure 4:**
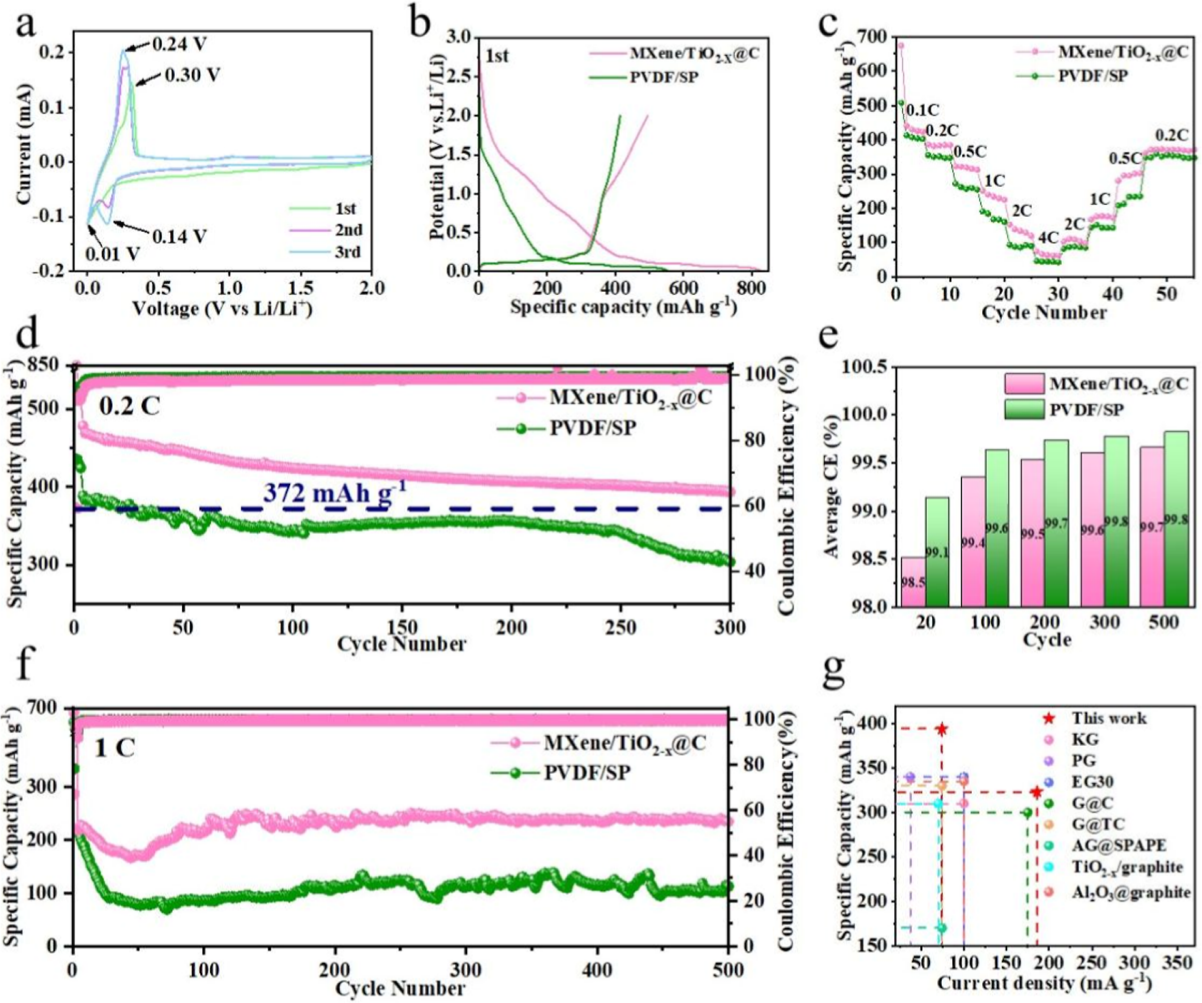
(a) CV of the AEA-G electrode during the first three cycles at a scan rate of 0.1 mV s^–1^. (b) Voltage-capacity curves of the AEA-G and PS-G electrodes in the first cycle. (c) Rate performance, (d) long-term cycling performance at 0.2C, (e) average CE, and (f) long-term cycling performance at 1C of the AEA-G and PS-G electrode. Mass loading of all electrodes is 1.2 mg cm^–2^. (g) Comparison of the electrochemical performance of the AEA-G electrode with graphite electrodes reported in the literature.^35–42^

The electrochemical performance of the electrodes was further evaluated by assembling coin cells. Graphite was regarded as the active material for calculating the specific capacities of all the electrodes. The voltage–capacity curves of the batteries for the first cycle are shown in Figure 4b. The AEA-G electrode delivers a high initial discharge capacity of 826 mA h g^–1^ and charge capacity of 494 mA h g^–1^, along with an initial Coulombic efficiency (ICE) of 59.8%. In contrast, the discharge and charge capacity of the PS-G electrode is 552 mA h g^–1^ and 414 mA h g^–1^, respectively, with an ICE of 75.0%. The larger irreversible capacity of the AEA-G electrode was caused by the high specific areas of MXene and TiO_2–*x*_@C, which consumed more electrolyte to form the SEI layer. Despite this, both the discharge and charge capacities were much higher than those of conventional graphite electrodes. The charge and discharge curves of the AEA-G electrode at 0.2C in different cycles indicate good reversibility during the charge–discharge cycling process (Figure S18).

Figure 4c compares the performance rate of the electrodes at various current densities. Upon increasing the current density stepwise from 0.1 to 4C, the AEA-G electrode provided capacities of 441, 386, 323, 252, 152, and 75 mA h g^–1^ and recovered to a capacity of 373 mA h g^–1^ when returned to 0.2C, demonstrating favorable reversibility and structural stability. In contrast, the capacity of the PS-G anode is much lower at all current densities. The AEA-G electrode displayed an initial discharge capacity of 478 mA h g^–1^ at 0.2C, as shown in Figure 4d (activated at 0.1C for three cycles). After 300 cycles, a capacity of 394 mA h g^–1^ can still be maintained, which is significantly higher than that of the PS-G electrode and the theoretical capacity of graphite. This is because MXene and TiO_2–*x*_@C contribute extra capacity in the AEA-G electrode. At a current density of 1C, an impressive capacity of 236 mA h g^–1^ was obtained after 500 cycles, which was markedly higher than that of the PS-G electrode (113 mA h g^–1^) (Figure 4f). The average Coulombic efficiency was 99.7%, indicating good reversibility (Figure 4e). Furthermore, under the same load mass, the compaction density of the AEA-G electrode was greater than that of the PS-G electrode. Therefore, the AEA-G electrode enhances the volumetric energy density of the battery.^34^ In Figure 4g, we carefully compare the battery performance of the AEA-G electrode with recent studies on graphite anodes (Table S1),^35–42^ further confirming the advantages and novelty of the AEA-G electrode over previously reported works.

### Kinetic Analysis

Electrochemical impedance spectroscopy (EIS) was performed to evaluate the kinetics of Li^+^ transport through the electrode/electrolyte interface. As shown in Figure 5a, the AEA-G electrode displays the smallest diameter among the semicircles in the high-frequency regions, suggesting the most favorable interfacial Li^+^ transportation dynamics along the SEI. Equivalent circuits were used to fit the electrochemical impedance spectra (Figure S19). The high- and intermediate-frequency areas indicate the electrolyte solution resistance (*R*_e_), solid electrolyte interphase resistance (*R*_SEI_), and charge transfer resistance (*R*_ct_), and the low-frequency areas correspond to the Warburg impedance (*W*).^43^ Figure 5b,c shows the fitted *R*_SEI_ and *R*_ct_ values for the AEA-G and PS-G electrodes, respectively. The *R*_SEI_ value of the AEA-G electrode is 4 Ω, much lower than that of PS-G electrode (9 Ω). In addition, the AEA-G electrode had a smaller *R*_ct_ value, which was less than half that of the PS-G electrode. The enhanced reaction kinetics of the AEA-G electrode could be attributed to the robust conductive network formed by the cooperation of the TiO_2–*x*_@C additives and MXene binders inside the electrode configuration. The aged AEA-G electrode demonstrated a smaller resistance than the PS-G electrode after 100 and 500 cycles, as shown in Figure 5a. The corresponding *R*_SEI_ and *R*_ct_ values of the fitted EIS curves (Figure 5b,c) show that the AEA-G electrode has the lowest interphase and charge transfer resistances. Besides, the AEA-G electrode undergoes a slight increase of merely 2 Ω in *R*_SEI_ from the 100th cycle to the 500th cycle, indicating a quite stable electrode/electrolyte interface.

**Figure 5:**
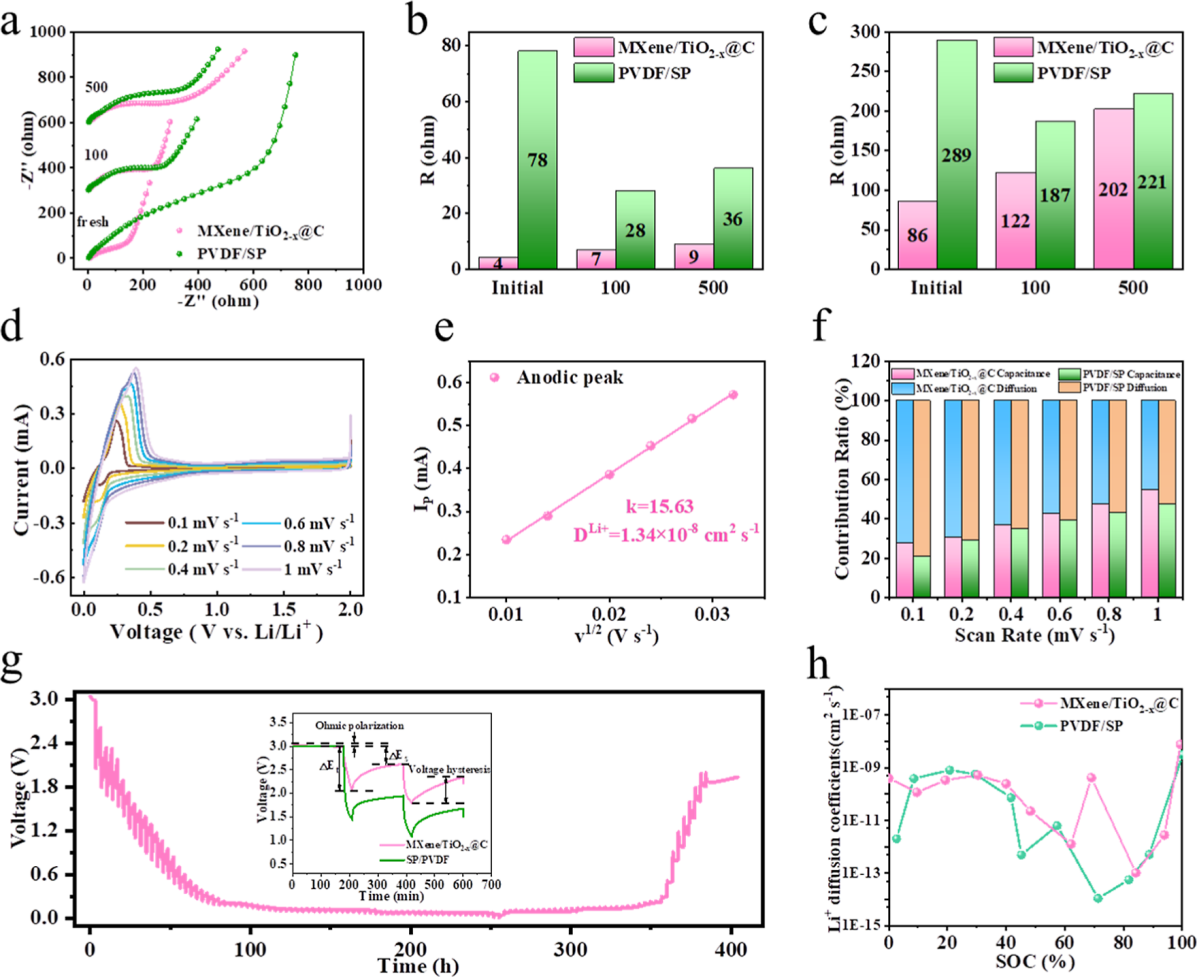
(a) EIS curves. (b) *R*_SEI_ statistics and (c) *R*_ct_ statistics of the AEA-G and PS-G electrodes in different cycles. (d) CVs obtained at different scanning rates. (e) Relationship between the peak current and scan rate of the AEA-G electrode. (f) Contribution ratios of the capacitive and diffusion-controlled processes at different scanning rates for the AEA-G and PS-G electrodes. (g) GITT curves of the AEA-G electrode. (h) Li^+^ diffusion coefficients for the AEA-G and PS-G electrodes during the delithiation process.

The Li^+^ diffusion characteristics of the AEA-G electrode were further investigated by cyclic voltammetry at different scanning rates of 0.1–1.0 mV s^–1^ (Figure 5d). Even at different scans, the curves had similar shapes of oxidation and reduction peaks, indicating good kinetics and low polarization.

At different scanning rates, the peak cathode and anode currents (*I*_p_) are linearly related to the square root of the scanning rate, which can be used to characterize the Li-ion diffusion coefficient (*D*_Li^+^_) according to the classical Randles–Sevcik equation

1 $$I_{P}=\left(2.69\times 10^{5}\right)n^{1.5}AD_{{Li}^{+}}^{1/2}v^{1/2}C_{{Li}^{+}}$$

where *I*_p_ is the peak current, *n* is the number of charge transfers, *A* is the electrode area, *C*_Li^+^_ is the concentration of Li ions in the electrolyte, and *v* is the potential scanning rate.^44,45^ Based on the above equation, the slope of the linear relationship of the *I*_p_–*v* ^1/2^ diagram helps estimate the *D*_Li^+^_ of the electrode (Figure 5e). As shown in Figure 5e, the *D*_Li^+^_ of the AEA-G electrode calculated by the Randles–Sevcik equals 1.34 × 10^–8^ cm^2^ s^–1^.

Furthermore, owing to the ohmic resistance and the intrinsic electrochemical dynamics limit, the drift of the oxidation and reduction peaks increased with the scan rate. The relationship between the peak current (*i*) and scan rate (*v*) can be described as follows

2 $$i=av^{b}$$

The value of b was determined from the slopes of the log­(*v*) and log­(*i*) plots. This value reveals the dominant storage mechanism: *b* = 0.5 represents diffusion control and Faraday insertion, whereas *b* = 1 represents capacitance control owing to the linear relationship between the capacitance current and sweep rate. Figure S20 shows the relationship between log­(*v*) and log­(*i*), from which the values of the cathode and anode peaks are determined to be 0.71 and 0.54, respectively. This indicated that the ion storage dynamics of the AEA-G electrode were governed by both diffusion and capacitance. To further quantify the contributions of the capacitive and diffusion-control behavior to the capacity, the following equation was used

3 $$i=k_{1}v+k_{2}v^{1/2}$$

Constants *k*_1_ and *k*_2_ were determined by fitting the current values at different scanning rates and voltage points. In this equation, *k*_1_ *v* corresponds to the capacitance contribution and *k*_2_ *v* ^1/2^ corresponds to the diffusion control contribution.^46^

At different scan rates of 0.1–1.0 mV s^–1^, the capacitive-controlled contributions of the AEA-G electrode are 27%, 30%, 37%, 43%, 48%, and 54%, respectively (Figure 5f). The capacitive controlled-contribution increased with the scan rate and surpassed the diffusion-controlled contribution at 1.0 mV s^–1^, which also explains the vigorous dynamics at high current densities.

Galvanostatic intermittent titration technique (GITT) measurements were performed to investigate the dynamic behaviors of the electrodes. Figure 5g shows that the AEA-G electrode exhibited smaller ohmic polarization and voltage hysteresis, indicating faster kinetics than those of the PS-G electrode. The diffusion coefficient of Li^+^ was calculated using the simplified Fick’s second law equation

4 $$D=\frac{4}{\pi \tau }{\left(\frac{m_{B}V_{M}}{M_{B}S}\right)}^{2}{\left(\frac{{\Delta E}_{S}}{{\Delta E}_{\tau }}\right)}^{2}$$

where τ is the duration of the current pulse, Δ*E*_S_ is the quasi-thermodynamic equilibrium potential difference before and after the current pulse, Δ*E*_τ_ represents the potential difference during the relaxation of current pulse, *m*_B_, *V*_M_, *M*_B_ and *S* are the active mass, molar volume, molar mass and active surface area of the electrode, respectively.^47^ The calculated charge mobility results show that the AEA-G electrode (Figure 5h) has a higher Li^+^ diffusion rate than the PS-G electrode in most states during the discharge process, which is consistent with its satisfactory rate capability. Table 1 presents a comparison between the performances of the AEA-G and PS-G electrodes. The AEA-G electrode exhibits better cycling performance, rate capability, and lithium-ion diffusion coefficient than the PS-G electrode.

**Table 1:** Comparison of the Different Parameters of the AEA-G and PS-G Electrodes

parameter	AEA-G	PS-G
ICE (0.2C)	59.8%	75.0%
0.2C (after 300 cycles)	394 mA h g^–1^	304 mA h g^–1^
ICE (1C)	67.3%	78.4%
1C (after 500 cycles)	236 mA h g^–1^	113 mA h g^–1^
0.1C	441 mA h g^–1^	420 mA h g^–1^
0.2C	386 mA h g^–1^	378 mA h g^–1^
0.5C	323 mA h g^–1^	290 mA h g^–1^
1C	252 mA h g^–1^	210 mA h g^–1^
2C	152 mA h g^–1^	116 mA h g^–1^
4C	75 mA h g^–1^	59 mA h g^–1^
*D*_Li^+^_	1.34 × 10^–8^ cm^2^ s^–1^	1.24 × 10^–8^ cm^2^ s^–1^

### In Situ and Ex Situ Characterization

In situ X-ray diffraction was performed to monitor the structural evolution of graphite during the lithiation and delithiation processes (Figure 6a). Graphite underwent a series of solid-solution phase transitions from primary graphite to stage-4, a biphasic transition from stage-4 to stage-3, a solid-solution phase transition during stage-3, a biphasic transition from stage-3 to stage-2, and a biphasic transition from stage-2 to stage-1, respectively.^48^ However, at the same discharge rate, the PS-G electrode hardly reach stage-1 (Figure 6b). This indicates that the graphite was not fully lithiated during the storage of lithium ions in the PS-G electrode. These results indicate that the lithium storage capacity of graphite was fully utilized during the lithiation and delithiation of the AEA-G electrodes, demonstrating the high Li^+^ activity of AEA-G.

**Figure 6:**
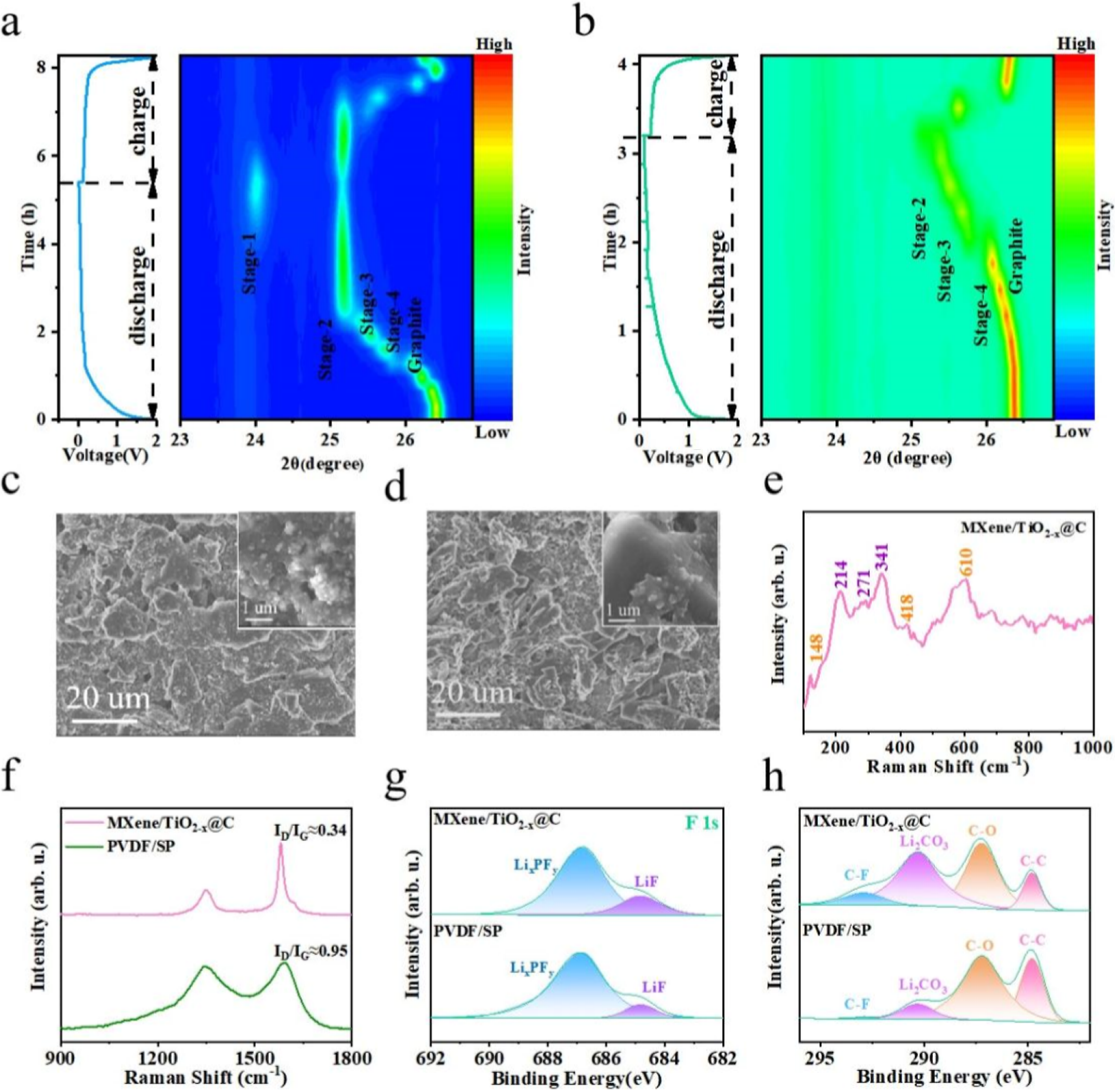
In situ XRD patterns of (a) AEA-G electrode and (b) PS-G electrode recorded during lithiation and delithiation processes. SEM image of (c) AEA-G electrode and (d) PS-G electrode after 100 cycles of charge and discharge. (e) Raman spectra of AEA-G electrode after 100 cycles of charge and discharge. (f) Raman spectra, (g) F 1s and (h) O 1s XPS spectrum of AEA-G electrode and PS-G electrode after 100 cycles of charge and discharge.

An SEM image of the AEA-G electrode in its initial state is shown in Figure S1. MXene and TiO_2–*x*_@C can be observed to be uniformly distributed around the graphite. The SEM images of the PS-G electrode in the initial state are shown in Figure S23. The binders and SP were uniformly distributed on the graphite. Figure 6c,d show the SEM images of the AEA-G and PS-G electrodes after 100 charge/discharge cycles. The AEA-G electrode maintained its integrity after cycling, whereas the binder and conductive additives in the PS-G electrode were dispersed, which was not conducive to maintaining the electrochemical capacity in subsequent charge and discharge cycles. This indicates that the AEA-G electrode has better stability during the charge and discharge processes.

The Raman spectrum of the AEA-G electrode in the initial state shows peaks corresponding to TiO_2–*x*_@C and MXene (Figure S24). After 100 charge–discharge cycles, the Raman curve also showed peaks corresponding to TiO_2–*x*_@C and MXene (Figure 6e). This indicates that the MXene and TiO_2–*x*_@C structures in the AEA-G electrode maintained their integrity after 100 charge and discharge cycles. Figure S25 shows the Raman spectra of the initial states of the AEA-G and PS-G electrodes. The *I*_D_/*I*_G_ ratio of the AEA-G electrode was 0.26, which was smaller than that of the PS-G electrode. This indicates that, in the initial state, the graphite AEA-G electrode was structurally intact and had minor defects. The aged AEA-G and PS-G electrodes were analyzed by Raman spectroscopy after 100 cycles (Figure 6f). The change in the I_D_/I_G_ ratio of the AEA-G electrode was less than that of the PS-G electrode, indicating that the surface defects of the AEA-G electrode increased less, and the graphite structure was maintained well after 100 cycles, which is more conducive to the insertion and removal of lithium ions.

To further analyze the compositional changes in the electrodes after cycling, XPS was performed. The F 1s XPS profiles of the AEA-G and PS-G electrodes after 100 charge–discharge cycles are shown in Figure 6g. The peak at 686.8 eV can be attributed to Li_*x*_PF_*y*_, and the peak at 684.8 eV can be attributed to LiF.^47^ The LiF content of the AEA-G electrode was higher than that of the PS-G electrode. A higher LiF content is conducive to stabilizing the electrode/electrolyte interface. This indicates that the AEA-G electrode was superior to the control sample in terms of ion transport. The C 1s XPS profiles of the AEA-G and PS-G electrodes after 100 charge–discharge cycles are shown in Figure 6h. The peak at 284.8 eV mainly represents C–C, the characteristic peak at 287.2 eV corresponds to C–O, the characteristic peak at 289.4 eV corresponds to Li_2_CO_3_, and the characteristic peak at 292.9 eV corresponds to C–O.^49^ The AEA-G electrode exhibited a stronger Li_2_CO_3_ signal than the PS-G electrode, which facilitated the transport of lithium ions at the electrode–electrolyte interface.

## Conclusions

In summary, we proposed a method for developing an all-electrochemically active graphite electrode by manipulating the Li^+^ activity of the inactive components, thereby enhancing the energy and power densities of the entire electrode. We demonstrated that by maintaining the colloidal state, MXene nanosheets can be employed as effective binders because of their abundant surface hydrogen bonding. Through nanostructures, carbon coatings and defect engineering, TiO_2–*x*_@C has excellent electrical conductivity that far exceeds that of commercial conductive additives, allowing for the construction of efficient conductive networks inside electrodes. MXene and TiO_2–*x*_@C demonstrated the ability to reversibly store lithium ions without significant structural degradation. Consequently, the as-prepared all-electrochem-active graphite electrode demonstrates a remarkable specific capacity of 394 mA h g^–1^ after 300 cycles. Furthermore, the rate capability was significantly enhanced because both the binders and conductive additives were electrochemically active toward electrons and Li^+^. In addition, MXene and TiO_2–*x*_@C act as substitutes for traditional additives, each with its own advantages and disadvantages in terms of cost, scalability, and environmental impact. Both require optimization through green synthesis (such as fluorine-free etching and waste liquid recycling) and large-scale technologies to enhance their practicality. Finally, this study is expected to advance the development of high-energy-density and high-power-density batteries through the manipulation of ion activity in binders and conductive additives, achieved by exploring materials or modifying existing commercial materials.

## Supplementary Material



## Notes

Experimental method, SEM image, Raman spectra, BET figure, HRTEM, SAED, XPS spectra, EPR spectra, CV curves, voltage-capacity curves, GITT curves, and a table comparing electrochemical performance (PDF)
