# Supplementary material for: All-Electrochem-Active Graphite Electrode Enabled by Manipulating Li+ Activity of Inactive Components for High-Energy Batteries
Source: ACS Appl Energy Mater. 2025 Jun 9;8(12):8277–87. doi: 10.1021/acsaem.5c00794 (PMC12188515; doi:10.1021/acsaem.5c00794)
Supplement: Supplementary file 1 [file ae5c00794_si_001.pdf]

Supporting Information

All-Electrochem-Active Graphite Electrode Enabled by Manipulating Li^+ Activity of Inactive Components for High-Energy Batteries

Junjin Zhang, ▿ Qitao Shi, ▿ Chen Lu, Xiangqi Liu, Jiaqi Wang, Cheng Zhang, Zhipeng Wang, Luwen Li, Alicja Bachmatiuk, Yanbin Shen,* Ruizhi Yang,* and Mark H. Rümmeli**

METHODS

MXene binders is prepared as follows: In general, 3.2 g of LiF (AR, Macklin) was added to 40 mL 9 mol L^{-1} HCl (AR, Yonghua) , transferred to an oil bath and stirred at 40 ℃ for 15 minutes until a transparent solution is formed. Then, 2 g Ti_3AlC_2 (<100 μm, Aladin) Max phase was added slowly under magnetic stirring and reacted at 40 ℃ for 48 h at a rotating speed of 650 rpm. After the reaction, the liquid was transferred to the centrifugal tube, and the excess LiF was washed with 100 mL 2 mol L^{-1} HCl. The supernatant was then poured out, 40 ml of DI water was added to the suspension, and then violently shaken, followed by another round of centrifugation at 3500 rpm for 5 minutes. This washing process was repeated 4 times until the pH of the upper liquid is ~6. The upper liquid was collected by ultrasound for one hour and centrifuged at 3500 rpm for 30min. The collected upper liquid was a

single layer of MXene colloid. The concentration of MXene was determined to be 22 mg mL^{-1} by filtration.

TiO_{2-x}@C conductive additives are prepared as follows: Anatase type TiO_2 (5 – 10 nm, Macklin) was heated in a tube furnace under an argon-hydrogen mixture atmosphere at 500 ℃ for 5 h to obtain TiO_{2-x}. Then, the obtained TiO_{2-x} was placed in a tube furnace, and heated to 900 ℃ under argon-hydrogen mixture atmosphere with acetonitrile (AR, Aladin) as carbon source at the vent for 2 hours. Then, TiO_{2-x}@C was milled in a planetary ball mill at 100 rpm for 5 hours.

Electrode preparation. Using MXene viscous ink as binders, TiO_{2-x}@C as conductive additives, and graphite (Canrd) as active substance, the slurry was prepared by stirring. The prepared electrode slurry was coated on copper foil with a scraper, and the AEA-G electrode was prepared by cutting the dry electrode. The mass ratio of the graphite/MXene/TiO_{2-x}@C electrode was controlled at 80:10:10. The load mass to area ratio was 1 mg cm^{-2}. To highlight the advantages of AEA-G electrode, PS-G electrode has also been prepared. (1) Based on graphite/PVDF/SP electrode, the mass ratio of the electrode was controlled to 80:10:10. The load mass to area ratio was 1 mg cm^{-2}.

Electrochemical characterization. The battery pack was assembled in an Ar-filled glove box where water and oxygen levels are below 0.01 ppm. In the assembled a half cell, a 16 mm diameter Li foil was used as the counter electrode, and a Celgard 2500 PP film was used as the separator. The electrolyte was a mixed solvent composed of 1

M $LiPF_6$ in EC: DEC: DMC (1:1:1 by vol) , with 5 wt% FEC and 2 wt% VC additives, purchased from Canrd corporation. Long-term constant current cycle performance and rate performance were tested with the NEWARE BTS 80 programmable battery test system. The potential ranges from 0 V to 2 V with respect to Li/Li^+. EIS and cyclic voltammetry were performed using CHI 660 E Electrochemical Workstation (CH Instruments, Shanghai).

Material characterization: The morphology of the materials was observed by SEM (Hitachi, SU8010). To further investigate the structural details of the material, the material was characterized with transmission electron microscopy (TEM, FEI, Titan Themis Cued G2 300) at an accelerated voltage of 300 kV. The phase composition of the material at 2θ angles from 5° to 80° was analyzed by X-ray diffractometer (Bruker D8 Advance), irradiated with Cu Kα (λ=1.5406 Å). The defect density was characterized by confocal Raman spectrometer (HR Evolution). The specific surface area and pore size distribution were analyzed by Bruner-Emmett-Teller (BET) Micromeritics (ASAP 2460). The elemental compositions and valence states of the materials were characterized by Al-K-α monochromatic X-ray photoelectron spectroscopy with scanning range from 1 eV to 1500 eV. Powder resistivity tester (PRC 3100) is used to measure the electrical conductivity of the material.

Supporting Figures

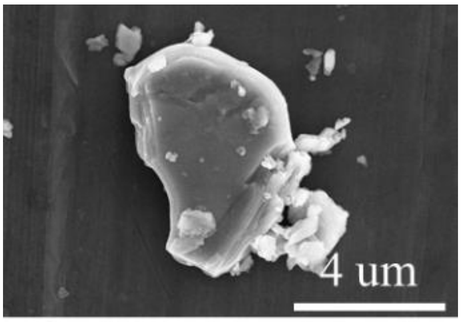

Figure S1. SEM image of Ti_3AlC_2.

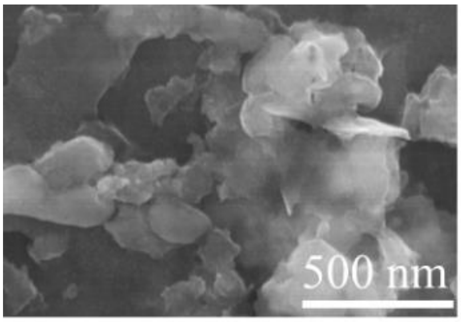

Figure S2. SEM image of freeze-dried MXene.

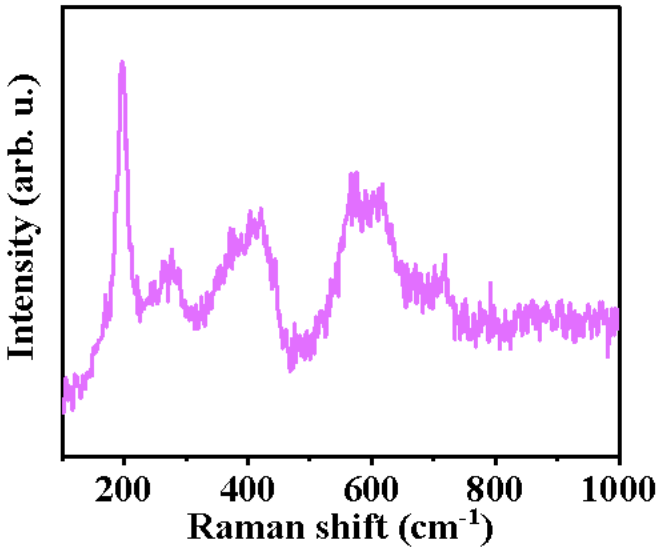

Figure S3. Raman spectra of MXene.

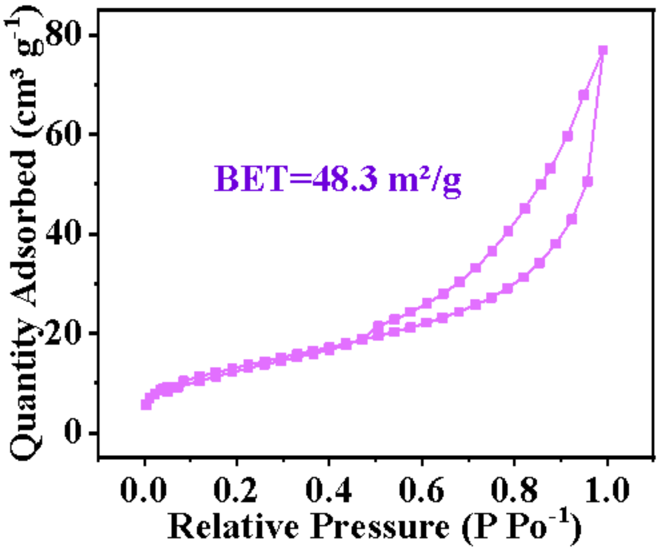

Figure S4. BET figure of MXene.

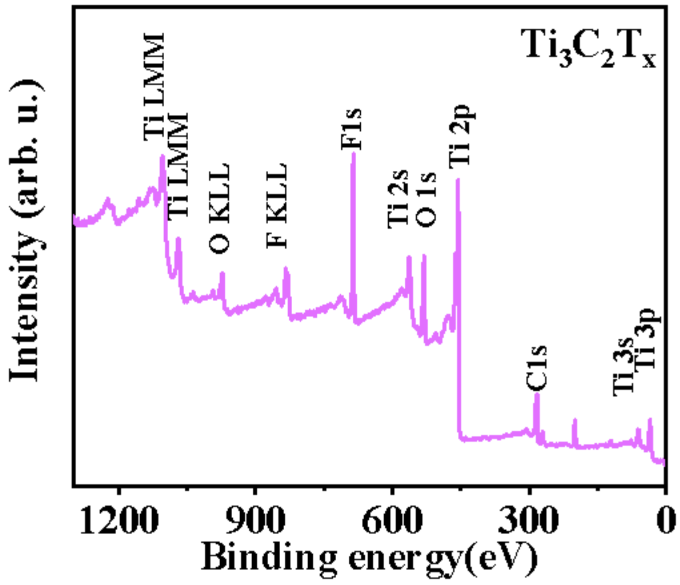

Figure S5. XPS survey spectrum of MXene.

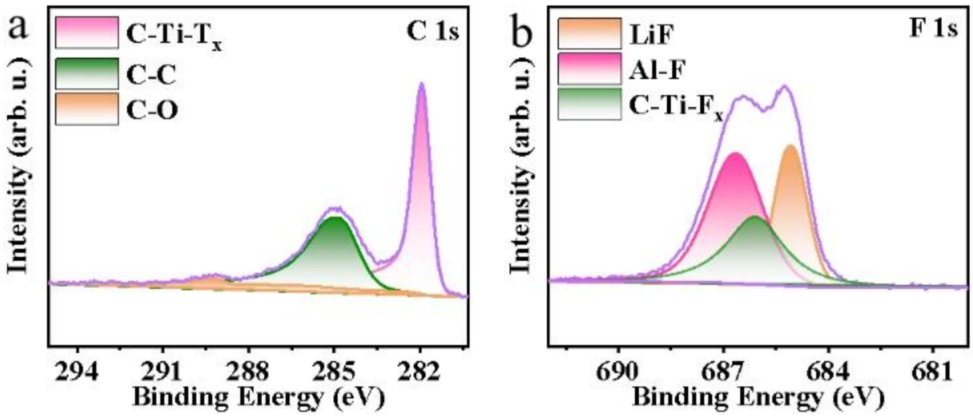

Figure S6. a) C 1s and b) F 1s XPS spectrum of MXene.

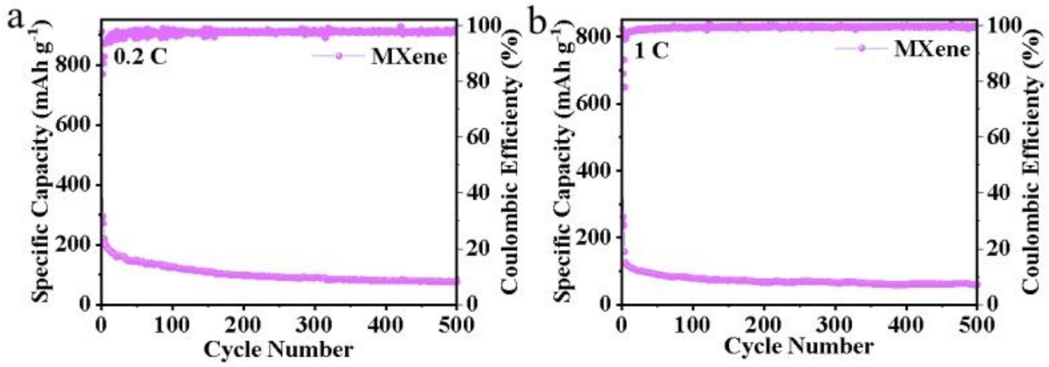

Figure S7. Cycling performance comparison of MXene electrode at a current rate of a) 0.2 C and b) 1 C.

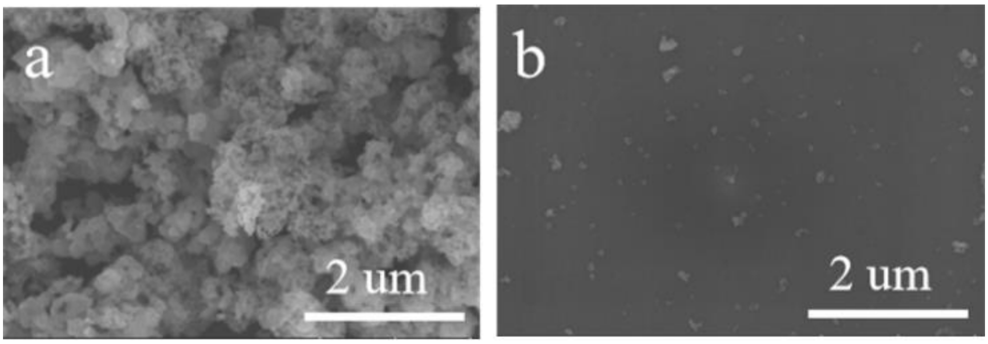

Figure S8. SEM image of a) TiO_2 and b) TiO_{2-x}@C.

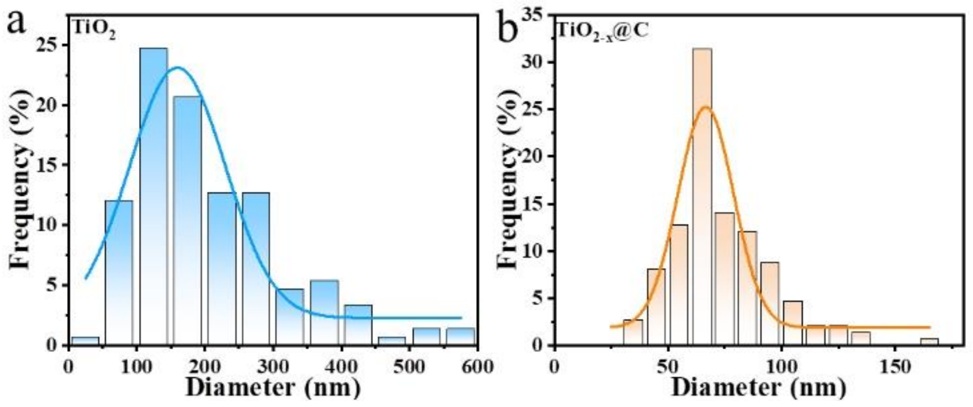

Figure S9. Diameter distributions of a) TiO_2 and b) TiO_{2-x}@C.

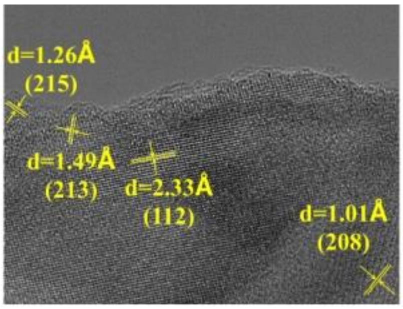

Figure S10. HRTEM of TiO_{2-x}@C.

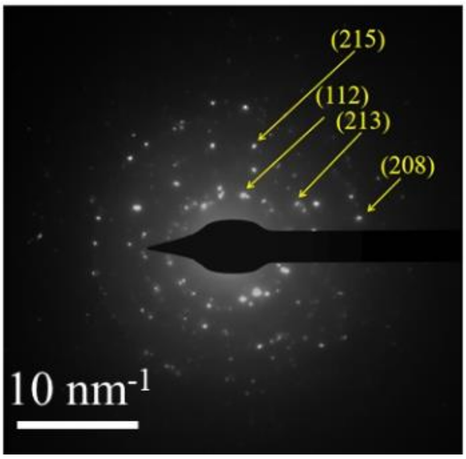

Figure S11. SAED of TiO_{2-x}@C.

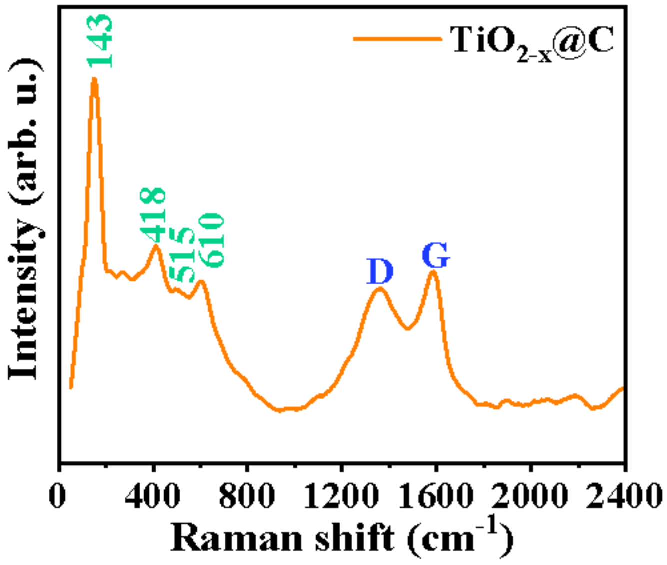

Figure S12. Raman spectra of TiO_{2-x}@C.

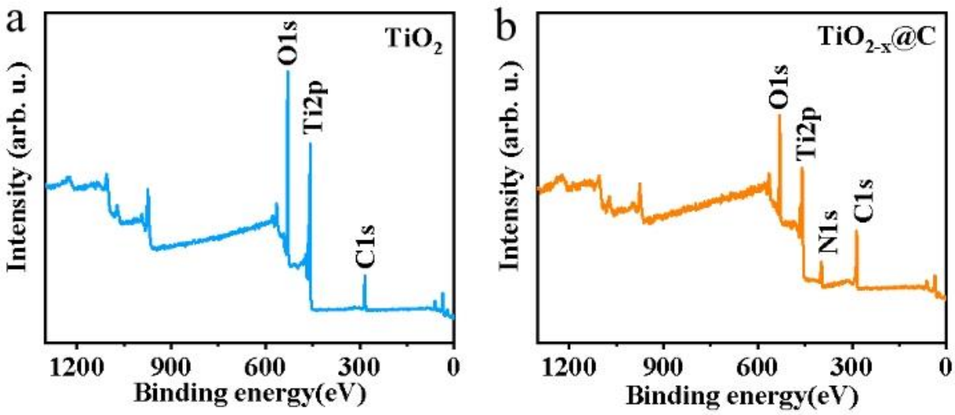

Figure S13. XPS survey spectrum of a) TiO_2 and b) TiO_{2-x}@C.

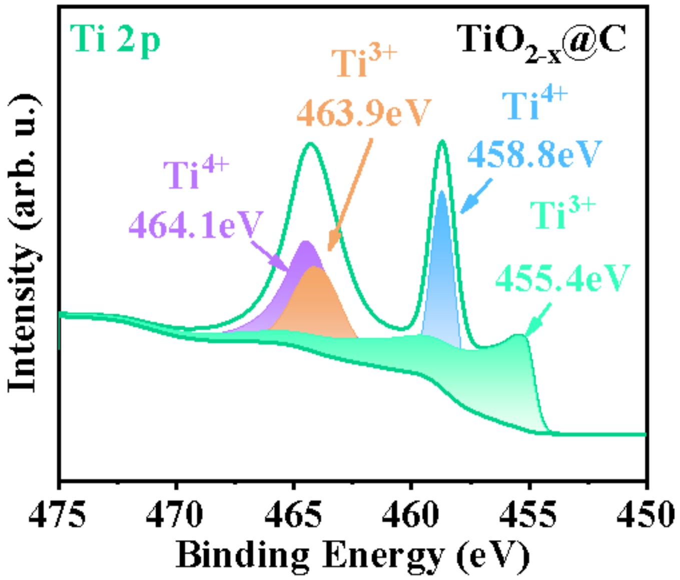

Figure S14. Ti 2p XPS spectrum of TiO_{2-x}@C.

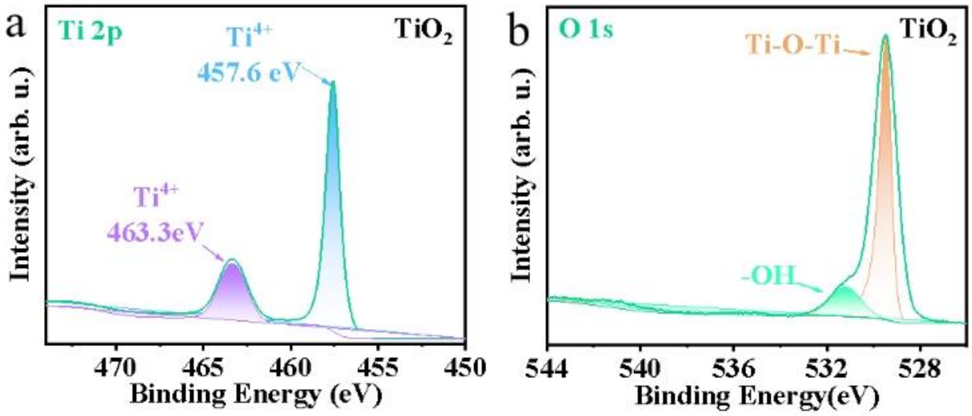

Figure S15. a) Ti 2p and b) O 1s XPS spectrum of TiO_2.

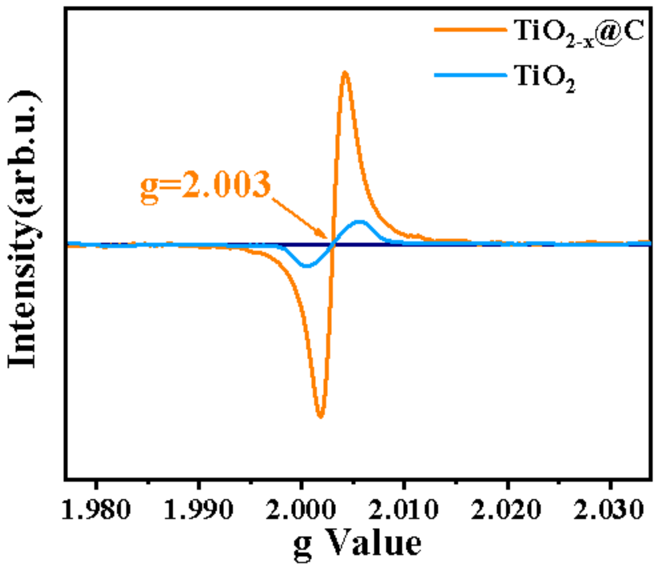

Figure S16. EPR spectra of TiO_{2-x}@C and TiO_2.

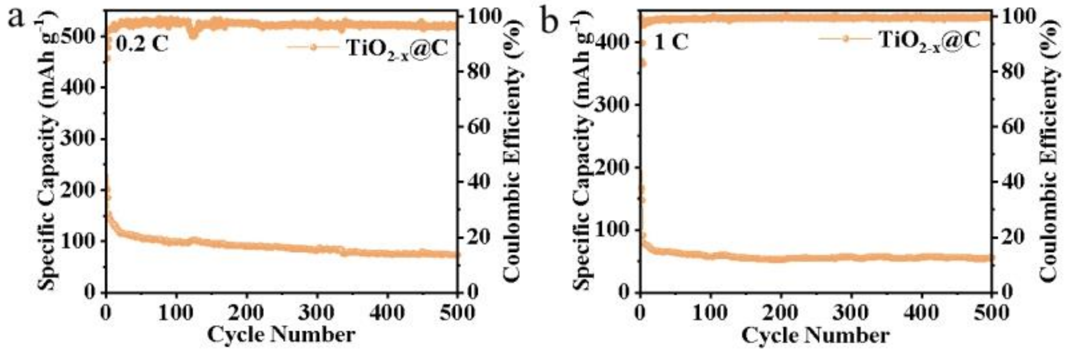

Figure S17. Cycling performance comparison of TiO_{2-x}@C electrode at a) 0.2 C and b) 1 C.

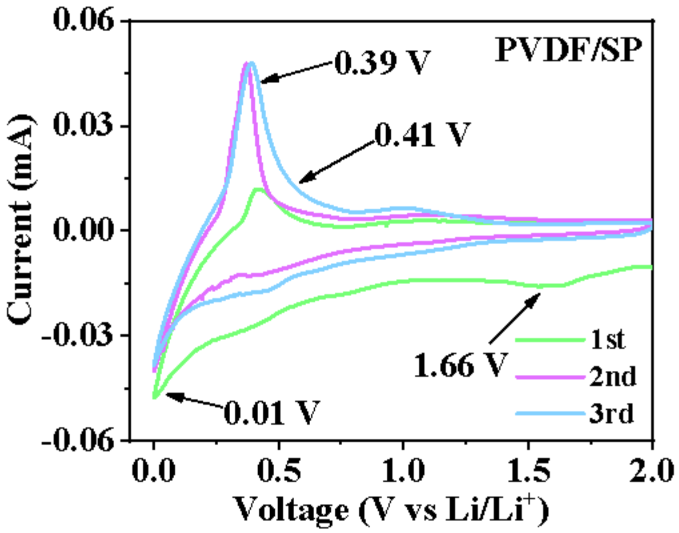

Figure S18. Cyclic voltammograms curves of PS-G electrode during the first three cycles at the scan rate of 0.1 mV s^{-1}.

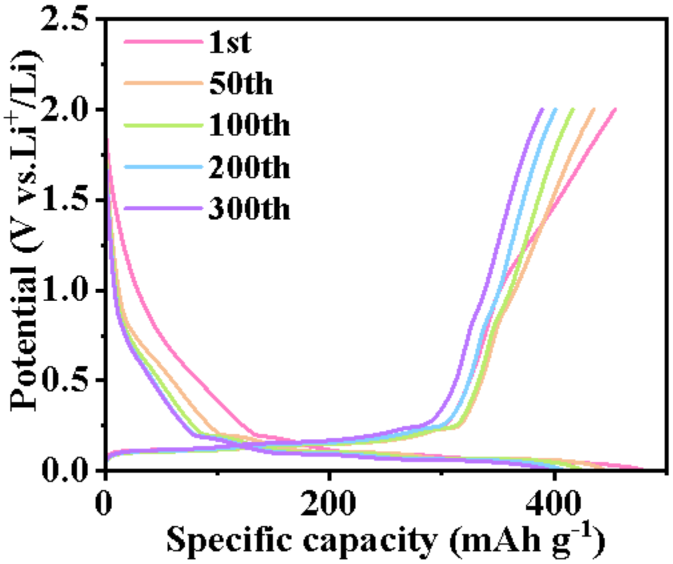

Figure S19. Voltage-capacity curves of AEA-G electrode at 0.2 C in different cycle.

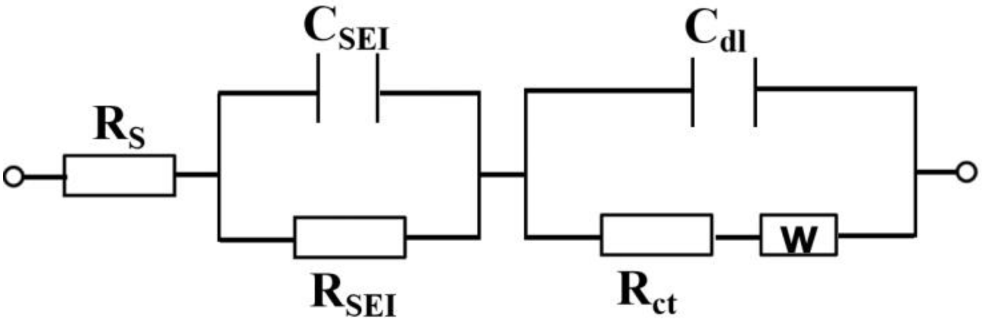

Figure S20. Equivalent circuit diagram for electrochemical impedance spectroscopy.

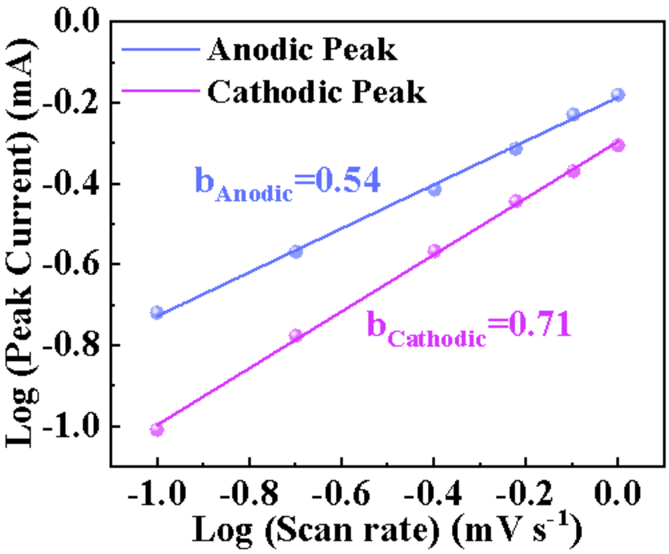

Figure S21. The relationship between the peak current and scan rate of AEA-G electrode.

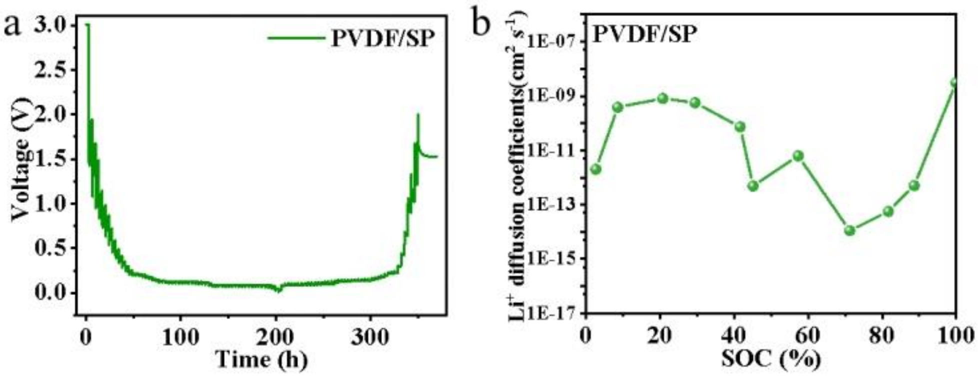

Figure S22. a) GITT curves of PS-G electrode for the discharging and charging process. b) Li^+ diffusion coefficients of PS-G electrode during delithiation process.

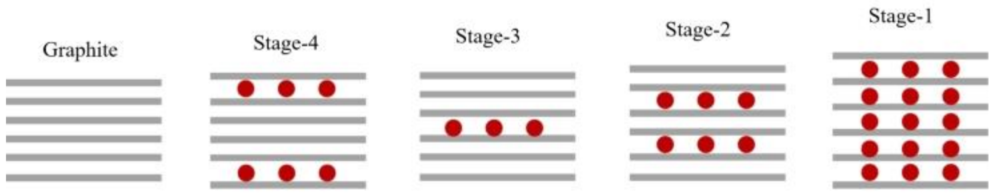

Figure S23. Schematic diagram of graphite at different stages of the lithiation process.

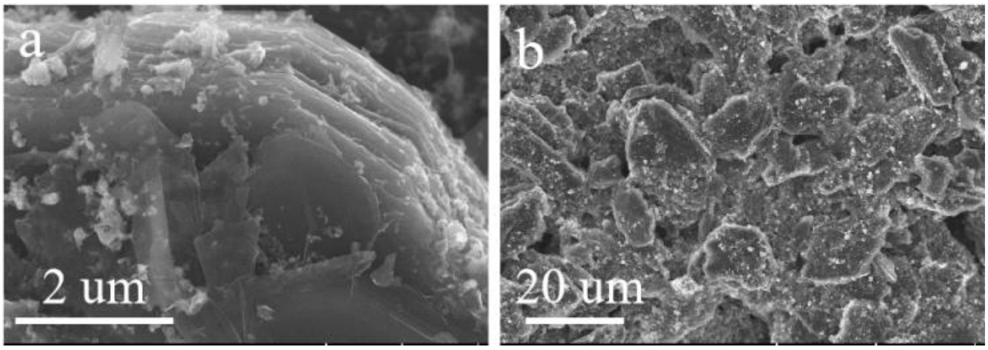

Figure S24. SEM image of AEA-G electrode in the initial state.

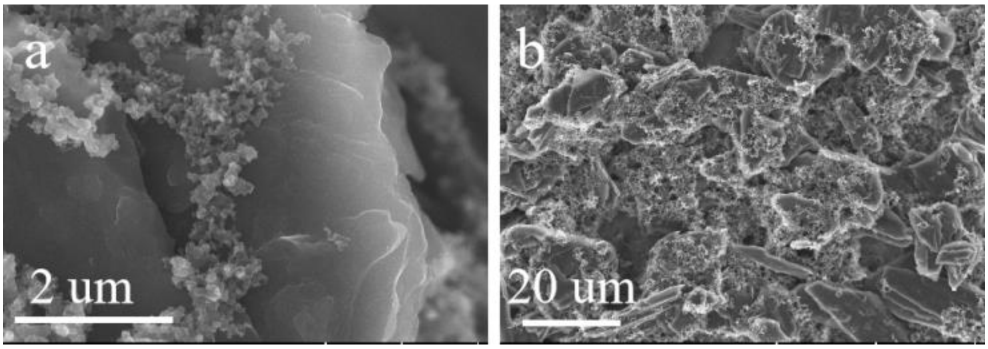

Figure S25. SEM image of PS-G electrode in the initial state.

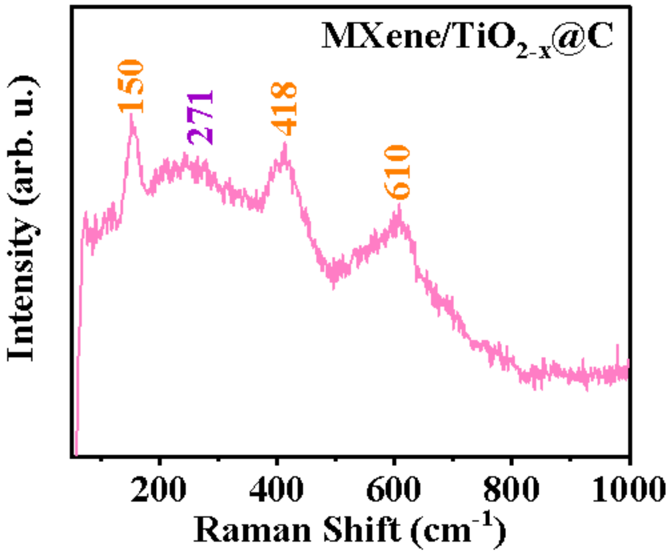

Figure S26. Raman spectra of AEA-G electrode in the initial state.

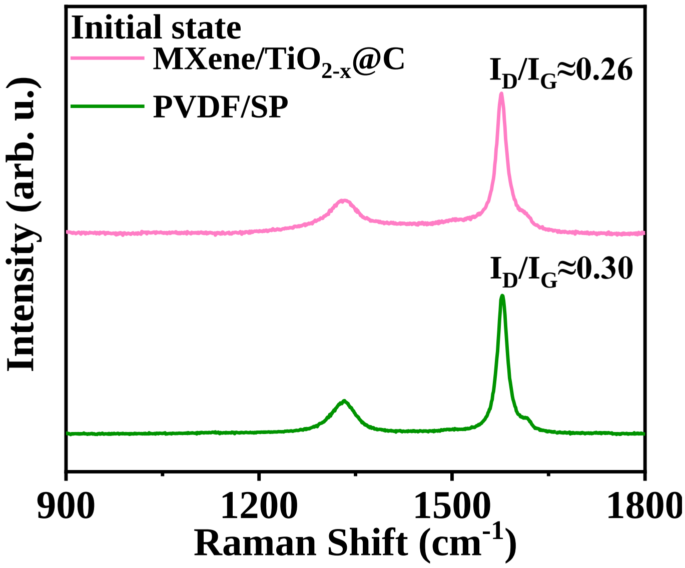

Figure S27. Raman spectra of AEA-G electrode and PS-G electrode in the initial state.

Electrode	Electrode composition	Current density (mA/g)	Capacity (mAh/g)	Number of cycle	Ref.
AEA-G electrode	Graphite:MXene:TiO_{2-x}@C=8:1:1	74.4	394	300	our work

AEA-G electrode	Graphite:MXene:TiO_{2-x}@C=8:1:1	186	323	-	our work
KG	KG:PVDF:SP=8:1:1	100	310	50	[1]
PG	PG:PVDF:SP=8:1:1	37.2	340	200	[2]
EG30	EG30:PVDF:SP=8:1:1	100	340	100	[3]
G@C	G@C:CMC:SP=90:5:5	175	300	-	[4]
G@TC	G@TC:PVDF:SP=8:1:1	74.4	330	-	[5]
AG@SPAPE-3%	AG@SPAPE-3%:CMC:SBR:SP=90:2.5:2.5:5	74.4	170	200	[6]
TiO_{2-x}/ graphite	TiO_{2-x}/ graphite:PVDF:SP=90:5:5	70	310	-	[7]
1 wt% Al_2O_3@graphite	1 wt% Al_2O_3@graphite:CMC:SBR=96:2:2	100	335	-	[8]

Table S1. Comparison the electrochemical performance between the AEA-G electrode and other reported graphite electrodes for LIB.

REFERENCES

(1) Kim, J.; Jeghan, S. M. N.; Lee, G. Superior fast-charging capability of graphite anode via facile surface treatment for lithium-ion batteries. *Microporous and Mesoporous Materials* **2020**, *305*, 110325.

(2) Shi, M.; Song, C.; Tai, Z.; Zou, K.; Duan, Y.; Dai, X.; Sun, J.; Chen, Y.; Liu, Y. Coal-derived synthetic graphite with high specific capacity and excellent cyclic stability as anode material for lithium-ion batteries. *Fuel* **2021**, *292*.

(3) Son, D.-K.; Kim, J.; Raj, M. R.; Lee, G. Elucidating the structural redox behaviors of nanostructured expanded graphite anodes toward fast-charging and high-performance lithium-ion batteries. *Carbon* **2021**, *175*, 187-201.

(4) Zhou, J.; Ma, K.; Lian, X.; Shi, Q.; Wang, J.; Chen, Z.; Guo, L.; Liu, Y.; Bachmatiuk, A.; Sun, J.; et al. Eliminating Graphite Exfoliation with an Artificial Solid Electrolyte Interphase for Stable Lithium-Ion Batteries. *Small* **2022**, *18* (15), e2107460.

(5) Cai, W.; Yan, C.; Yao, Y.-X.; Xu, L.; Xu, R.; Jiang, L.-L.; Huang, J.-Q.; Zhang, Q. Rapid Lithium Diffusion in Order@Disorder Pathways for Fast - Charging Graphite Anodes. *Small Structures* **2020**, 1 (1).

(6) Cai, K.; Xiang, C.; Wang, X.; Zhang, X.; Zhang, D.; Zheng, Z.; Jin, H.; Li, X.; Li, L. In-situ polymerization of p-sulfonated allyl phenyl ether coated graphite electrode for Lithium ion battery. *Journal of Energy Storage* **2024**, 84.

(7) Rhee, D. Y.; Kim, J.; Moon, J.; Park, M.-S. Off-stoichiometric TiO_2--decorated graphite anode for high-power lithium-ion batteries. *Journal of Alloys and Compounds* **2020**, *843*.

(8) Kim, D. S.; Kim, Y. E.; Kim, H. Improved fast charging capability of graphite anodes via amorphous Al_2O_3 coating for high power lithium ion batteries. *Journal of Power Sources* **2019**, *422*, 18-24..
